# Manganese Neurotoxicity as a Stroke Mimic: A Case Report

**DOI:** 10.7759/cureus.37247

**Published:** 2023-04-07

**Authors:** Mohiudeen Alikunju, Nafeesathu Misiriyyah, Shaikh Sayeed Iqbal, Maria Khan

**Affiliations:** 1 Neurology, Dubai Academic Health Corporation, Dubai, ARE; 2 Medicine, Dubai Academic Health Corporation, Dubai, ARE; 3 Radiology, Rashid Hospital, Dubai, ARE; 4 Neurology, Rashid Hospital, Dubai, ARE

**Keywords:** chelation, welding, manganism, stroke mimic, manganese neurotoxicity

## Abstract

Manganese (Mn)-induced cerebral toxicity is a rare neurological condition that can present as a stroke mimic in high-risk populations.

We present a case of a 40-year-old male with no known comorbidities who was brought to the emergency department with complaints of nonprogressive slurred speech and left facial weakness for eight days. Further history revealed that he had been working as a welder in a steel factory for the past seven years without using proper personal protective equipment (PPE). On physical examination, an upper motor neuron (UMN) type weakness on the left side of his face and spastic dysarthria could be appreciated. Following a brain computed tomography (CT) scan that showed ill-defined hypodensities in the basal ganglia without any signs of a hemorrhage, he was admitted to the stroke unit for conservative management and further investigations. A magnetic resonance imaging (MRI) scan of the brain done later showed features of manganese deposition and absorption in the globus pallidus and corticospinal tracts, indicating a diagnosis of manganese-induced cerebral toxicity. His serum manganese levels obtained during admission were normal. He was managed conservatively with intravenous rehydration and was discharged after symptomatic improvement. He was counseled and educated regarding the importance of wearing protective equipment while at work to reduce further exposure to the metal. During his follow-up visit, his symptoms had considerably improved with proper adherence to workplace safety measures.

## Introduction

Hypermanganesemia-induced cerebral toxicity is one of the rare metal neurotoxic conditions [1]. It can have a varied clinical presentation ranging from a Parkinsonian-like syndrome, also called manganism, to neuropsychiatric symptoms and very rarely as a stroke mimic [2]. Diagnosis is based on a comprehensive approach consisting of a detailed history, physical examination, laboratory studies, and diagnostic imaging. Management is primarily focused on rehabilitation and reducing further exposure to the metal [3]. However, in severe cases, manganese (Mn) excretion can be expedited by the administration of chelating agents such as calcium disodium ethylenediaminetetraacetic acid (Ca-EDTA) [4].

## Case presentation

A 40-year-old male with no known comorbid conditions presented to the emergency department with complaints of slurred speech and left facial weakness for a duration of eight days. It was associated with heaviness of the tongue and difficulty swallowing that caused drooling of saliva from the left side of his mouth. The symptoms were nonprogressive, and no other neurological deficits or systemic complaints were reported.

He had no significant past medical or surgical history. However, he reported that he had been working as a steel welder in a factory for the past seven years without consistent usage of personal protective equipment (PPE) or a filtering face mask.

Physical examination revealed a vitally stable, slightly anxious young male in no apparent distress. He had a left-sided upper motor neuron (UMN) type facial weakness and spastic dysarthria with a normal higher mental function. All other cranial nerves were intact, and no gross dysfunction in strength, coordination, sensation, gait, or the autonomic system could be appreciated.

Laboratory tests obtained prior to admission are summarized in Table 1, which yielded normal results. An electrocardiogram showed normal sinus rhythm with no notable abnormalities.

**Table 1:** Biochemical tests obtained before admission. WBC: white blood cell, RBC: red blood cell, SARS-CoV-2 RNA PCR: severe acute respiratory syndrome-associated coronavirus 2 ribonucleic acid polymerase chain reaction

Laboratory test	Laboratory value	Normal reference range
Complete blood count		
WBC count	7.6 k/uL	3.6-11 k/uL
RBC count	5.34 MIL/uL	3.80-4.80 MIL/uL
Hemoglobin, blood	16.1 g/dL	12-15 g/dL
Hematocrit	47.3%	36%-46%
Platelet count	308 k/uL	150-410 k/uL
General biochemistry		
Calcium, serum	9 mg/dL	8.9-10.2 mg/dL
Glucose, serum	87 mg/dL	60-100 mg/dL
Lactic acid, serum	0.9 mmol/L	0.5-2.2 mmol/L
Creatinine, serum	0.8 mg/dL	0.5-0.9 mg/dL
Bilirubin, serum total	0.7 mg/dL	0-1.2 mg/dL
Alkaline phosphatase, serum	71 U/L	35-104 U/L
Alanine aminotransferase, serum	18 U/L	0-31 U/L
Total protein	8.4 g/dL	6.6-8.7 g/dL
Albumin	4.4 g/dL	3.4-4.8 g/dL
Gamma-glutamyl transferase, serum	23 U/L	8-61 U/L
Troponin	4 ng/L	<14ng/L
Low-density lipoprotein	110 mg/dL	<115 mg/dL
SARS-CoV-2 RNA PCR swab (nasopharynx)	Not detected	Not detected

A computed tomography (CT) scan of the brain showed ill-defined hypodensities in the basal ganglia without any signs of a hemorrhage or an ischemic infarction.

Due to his clinical presentation and the absence of specific changes on the CT scan, the patient was admitted to the stroke unit with a National Institutes of Health Stroke Scale (NIHSS) score of 4 for further evaluation. He was started on a prophylactic dose of aspirin 75 mg, intravenous fluids, and a mashed diet.

**Figure 1:**
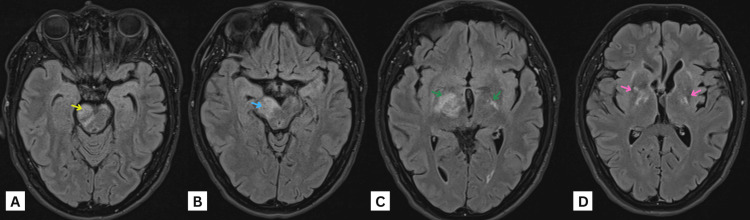
MRI of the brain axial FLAIR images at the level of the (A) pons, (B) midbrain, (C) upper midbrain, and (D) basal ganglia showing bilateral, almost symmetrical, signal alteration in the form of T2-FLAIR high-signal intensity involving the globus pallidus (pink arrows), corticospinal tracts (green arrows), and corona radiata. It starts from the corona radiata superiorly at the level of lateral ventricles extending to cerebral peduncles of the midbrain (blue arrows) inferiorly, affecting the right side more at this level. Similar signal alteration is also noted at the right anterolateral side of the pons (yellow arrows). No diffusion restriction can be seen. MRI: magnetic resonance imaging, FLAIR: fluid-attenuated inversion recovery

**Figure 2:**
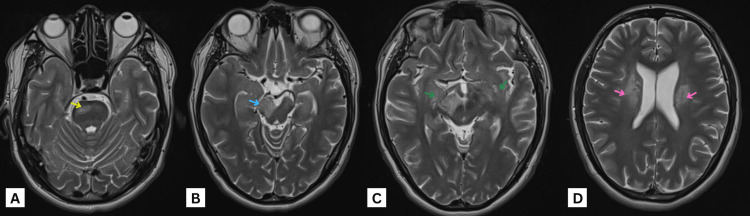
MRI of the brain axial T2 images at the level of the (A) pons, (B) midbrain, (C) upper midbrain, and (D) corona radiata, showing bilateral, almost symmetrical, signal alteration in the form of T2-FLAIR high-signal intensity involving the globus pallidus, corticospinal tracts (green arrows), and corona radiata (pink arrows). It starts from the corona radiata superiorly at the level of lateral ventricles extending to cerebral peduncles of the midbrain (blue arrows) inferiorly, affecting the right side more at this level. Similar signal alteration is also noted at the right anterolateral side of the pons (yellow arrows). No diffusion restriction can be seen. MRI: magnetic resonance imaging, FLAIR: fluid-attenuated inversion recovery

**Figure 3:**
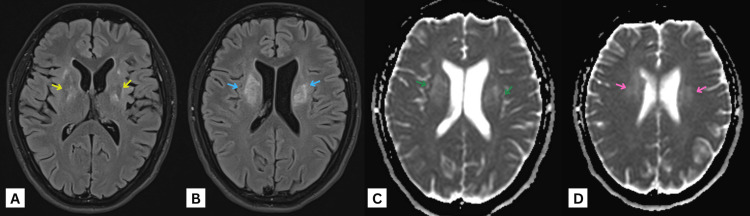
MRI of the brain axial FLAIR images at the level of the (A) basal ganglia and (B) corona radiata, and MRI of the brain axial ADC images at the level of the (C) corona radiata and (D) parietal cortex, showing bilateral, almost symmetrical, signal alteration in the form of T2-FLAIR high-signal intensity involving the globus pallidus (yellow arrows), corticospinal tracts, and corona radiata (blue, green, and pink arrows). It starts from the corona radiata superiorly at the level of lateral ventricles extending to cerebral peduncles of the midbrain (blue arrows) inferiorly, affecting the right side more at this level. Similar signal alteration is also noted at the right anterolateral side of the pons. No diffusion restriction can be seen. MRI: magnetic resonance imaging, FLAIR: fluid-attenuated inversion recovery, ADC: apparent diffusion coefficient

A magnetic resonance imaging (MRI) of the brain was done on the following day, which showed bilateral signal alteration in the form of T2 fluid-attenuated inversion recovery (FLAIR) hyperintensities involving the globus pallidus and corticospinal tract, extending superoinferiorly from the corona radiata to the cerebral peduncles of the midbrain, more extensive on the right side. These findings were consistent with his clinical presentation and neurological deficits. The basal ganglia and cortical gray matter were relatively spared, and no restricted diffusion was noted, effectively ruling out stroke as a differential diagnosis. All the above findings were indicative of a toxic encephalopathy, with manganese neurotoxicity being the most probable etiology owing to the characteristic MRI changes (Figures 1-3).

His serum manganese and other metal levels obtained were found to be within the reference range (Table 2), despite which the patient was diagnosed as a case of manganese neurotoxicity due to his occupation, clinical presentation, and characteristic imaging findings.

**Table 2:** Biochemical quantification of heavy metals done during admission.

Metal	Laboratory value	Normal reference range
Manganese, serum	0.9 mg/L	0.3-1.1 mg/L
Iron, serum	69 mg/dL	59-158 g/dL
Zinc, serum	81.4 mg/dL	46-150 mg/dL
Nickel, serum	<0.20 mg/L	0.05-1.05 mg/L
Arsenic, serum	4 mg/L	0-9 mg/L
Cadmium, serum	0.6 mg/L	0-1.2 mg/L
Mercury, serum	2.3 mg/L	0-14.9 mg/L

Over the course of his hospital stay, he received supportive care with intravenous hydration. Due to his normal serum manganese level, no chelation therapy was warranted. He was counseled regarding the importance of wearing personal protective equipment consistently, including a filtering face mask and a face shield while at work to prevent the worsening of his condition. The patient was discharged with a follow-up appointment in the outpatient neurology clinic after two weeks.

On his follow-up visit, he reported continuous improvement in the severity of his symptoms upon adherence to the protective measures at work.

## Discussion

Manganese is one of the heavy metals that are found in the human body in trace amounts. It is required in low concentrations for the proper functioning of various enzymes. Despite diet being the major extrinsic source of manganese, small particles can be inhaled, which serves as an occupational hazard to metal welders and miners [3,5].

Owing to the rarity of hypermanganesemia-induced neurotoxicity, no clear statistics have been reported regarding its prevalence in the general population. However, a study conducted in China found the prevalence of manganism to be around 0.5%-2% among ferromanganese and silicomanganese plant workers [6]. Several factors that increase the risk of developing hypermanganesemia and neurotoxicity have been identified. Newborns and infants are predisposed to developing this condition due to their immature excretory mechanisms and metabolic processes. Female gender, pregnancy, old age, a preexisting neurocognitive dysfunction or history of hepatic failure, prolonged total parenteral nutrition (TPN), and occupational exposure are all considered to be risk factors [7].

The exact pathophysiology of this phenomenon is unknown; however, it is hypothesized to be a multifactorial complex mechanism characterized by a “manganese mechanistic neurotoxic triad” comprising mitochondrial dysfunction with oxidative stress, protein trafficking and misfolding, and neuroinflammation [8]. Manganese is primarily transported to the brain, permeating the blood-brain barrier, but it can also be inhaled and carried through the olfactory pathway or brain-cerebrospinal fluid (CSF) barrier, putting welders like our patient at an increased risk for developing neurotoxicity. This metal is removed from the brain by a slow diffusion process that can cause accumulation [4,5]. Manganese transporter SLC30A10 is reportedly associated with hypermanganesemia when mutated. This protein has its highest levels of expression in the liver and basal ganglia, explaining the predilection of manganese accumulation in these areas when plasma levels are high [5].

Symptoms of manganese neurotoxicity can vary based on the duration of exposure to the toxin. Manganism, which is a distinct disorder that affects the extrapyramidal system, is the most common presentation characterized by motor disturbances associated with neuropsychiatric and cognitive disabilities. The earliest manifestation in these cases is reported to be manganese madness, a condition distinguished by emotional outbursts, compulsive laughter, depression, hallucinations, and sleep disturbances. As the psychosis wanes, abnormalities in the motor system with Parkinsonian features of bradykinesia, muscular rigidity, and postural instability predominate. Higher mental dysfunction with a relative sparing of the language component has also been described [2]. Very rarely, manganese neurotoxicity can present as a stroke mimic with only one such case of a female on chronic TPN use being published [1].

As a metal that is primarily excreted through bile, biochemical quantification of urinary or serum manganese does not correlate with the severity of the neurotoxicity, rendering it inaccurate in establishing a clear diagnosis. Samples from saliva, nails, and hair have all yielded inconclusive results [7].

The manganese content in erythrocytes, called Mn-RBC assay, is an effective biomarker for the detection of manganese in the brain. Studies have shown that higher levels of manganese in erythrocytes correlate to greater accumulation in the brain [9].

Brain imaging using an MRI shows the characteristic features of manganese deposition and absorption, the most common of which is the hyperintense T1/T2 signal changes, often seen in the globus pallidus, substantia nigra, and parietal and occipital cortex [10]. These changes are usually symmetrical and bilateral with cerebellar atrophy reported in severe cases. All these findings typical for manganese deposition were seen in our patient. Additionally, diffusion restriction changes, T1 basal ganglia hypointensities, or diffuse brain swelling might be present. Rarely, cerebral atrophy, globus pallidal necrosis, and cerebral hemorrhages could be found. Therefore, it is reasonable to infer that MRI changes have the sensitivity and specificity to reflect manganese accumulation in the brain [10,11].

The cornerstone of management is to reduce further exposure to manganese by transferring them out of the precipitating environment and administering a gastrointestinal cleanse. Most patients show significant and lasting improvements in eliminating the source and only warrant regular follow-up to ensure adequate recovery [3]. Nevertheless, chelation therapy might be required in severe poisoning with agents such as calcium disodium ethylenediaminetetraacetic acid (CaNa2EDTA), which enhances the excretion of manganese from the blood. A study conducted in 2014 concluded that despite the EDTA-mediated excretion of manganese in urine, no significant clinical improvement was noted in these subjects [4]. Several research have demonstrated variable results regarding the effectiveness of newer treatment strategies, such as the use of antioxidants including polyphenolic compounds, trolox, N-acetylcysteine, and 3-methyl-1-phenyl-2-pyrazoline-5-one (edaravone). Nonspecific cyclooxygenase inhibitors, selective cyclooxygenase-2 inhibitors, and adenosine triphosphate/diphosphate ratio protectors may exhibit anti-inflammatory responses in this condition. Other potential treatment modalities are steroid hormones, selective estrogen receptor modulators, and synthetic prostaglandin analogs, owing to their ability to maintain glutamate homeostasis, reversing the negative effects of GABAergic and glutamatergic projections in the basal ganglia that lead to the motor deficits characterizing Mn neurotoxicity [8,12].

The recommended level of Mn exposure among welders as per the American Conference of Governmental Industrial Hygienists (ACGIH) of 2016 is 0.2 mg/m^3^. Strict policies including the usage of welding equipment with inbuilt exhaust ventilation, robotic machinery, personal protective equipment (PPE) such as filtered masks and gloves, and periodic measurements of the air quality are essential to maintain these standards. Additionally, health education and regular assessment of the protective equipment quality and workers’ health status are of paramount importance with emphasis on prompt identification of the early manifestations of hypermanganesemia. Teamwork between governmental agencies and private institutions is required to ensure these measures are being adequately followed [13].

## Conclusions

Working as a welder for more than half a decade with no consistent usage of PPE, our patient developed manganese-induced cerebral toxicity at a young age despite the absence of other risk factors such as liver failure or chronic TPN use. Therefore, considering heavy metal neurotoxicity as a differential diagnosis in patients presenting with neurological deficits is important, especially in those with increased exposure to these metals. Effective excretion of manganese by targeting the specific transporter SLC30A10 can have a significant role in the future management of hypermanganesemia. More studies in high-risk populations are required to explain the effectiveness of this treatment option and report the prevalence and morbidity of this condition, as it can cause irreversible neurophysiological dysfunction.

## References

[1] Reinert JP, Garner M, Forbes L (2021). Hypermanganesemia-induced cerebral toxicity mimicking an acute ischemic stroke: a case report and review of overlapping pathologies. J Pharm Technol.

[2] Bouabid S, Tinakoua A, Lakhdar-Ghazal N, Benazzouz A (2016). Manganese neurotoxicity: behavioral disorders associated with dysfunctions in the basal ganglia and neurochemical transmission. J Neurochem.

[3] Chen P, Miah MR, Aschner M (2016). Metals and neurodegeneration. F1000Res.

[4] Crossgrove J, Zheng W (2004). Manganese toxicity upon overexposure. NMR Biomed.

[5] Lee EY, Flynn MR, Lewis MM, Mailman RB, Huang X (2018). Welding-related brain and functional changes in welders with chronic and low-level exposure. Neurotoxicology.

[6] Kwakye GF, Paoliello MM, Mukhopadhyay S, Bowman AB, Aschner M (2015). Manganese-induced parkinsonism and parkinson's disease: shared and distinguishable features. Int J Environ Res Public Health.

[7] Evans GR, Masullo LN (2023). Manganese toxicity. https://www.statpearls.com/point-of-care/24728.

[8] Harischandra DS, Ghaisas S, Zenitsky G, Jin H, Kanthasamy A, Anantharam V, Kanthasamy AG (2019). Manganese-induced neurotoxicity: new insights into the triad of protein misfolding, mitochondrial impairment, and neuroinflammation. Front Neurosci.

[9] Jiang Y, Zheng W, Long L (2007). Brain magnetic resonance imaging and manganese concentrations in red blood cells of smelting workers: search for biomarkers of manganese exposure. Neurotoxicology.

[10] Kim EA, Cheong HK, Choi DS, Sakong J, Ryoo JW, Park I, Kang DM (2007). Effect of occupational manganese exposure on the central nervous system of welders: 1H magnetic resonance spectroscopy and MRI findings. Neurotoxicology.

[11] Zheng W, Fu SX, Dydak U, Cowan DM (2011). Biomarkers of manganese intoxication. Neurotoxicology.

[12] Marreilha dos Santos A, Andrade V, Aschner M (2017). Neuroprotective and therapeutic strategies for manganese-induced neurotoxicity. Clin Pharmacol Transl Med.

[13] Kulshreshtha D, Ganguly J, Jog M (2021). Manganese and movement disorders: a review. J Mov Disord.

